# Supplementary material for: Oncolytic H-1 parvovirus binds to sialic acid on laminins for cell attachment and entry
Source: Nat Commun. 2021 Jun 22;12:3834. doi: 10.1038/s41467-021-24034-7 (PMC8219832; doi:10.1038/s41467-021-24034-7)
Supplement: Supplementary file 1 — Supplementary Information [file 41467_2021_24034_MOESM1_ESM.pdf]

Oncolytic H-1 parvovirus binds to sialic acid on laminins for cell attachment and entry

Amit Kulkarni[1,2], Tiago Ferreira[1], Clemens Bretscher[1], Annabel Grewenig[1], Nazim El-Andaloussi[1,3], Serena Bonifati[1,4], Tiina Marttila[1,2], Valérie Palissot[2], Jubayer A. Hossain[2,5,6], Francisco Azuaje[7,8], Hrvoje Miletic[5,6], Lars A. R. Ystaas[5], Anna Golebiewska[9], Simone P. Niclou[9], Ralf Roeth[10,11], Beate Niesler[10,11], Amélie Weiss[12,], Laurent Brino[12,] and Antonio Marchini[1,2*]

1: Laboratory of Oncolytic Virus Immuno-Therapeutics, German Cancer Research Center, Im Neunheimer Feld 242, 69120, Heidelberg, Germany

2: Laboratory of Oncolytic Virus Immuno-Therapeutics, Luxembourg Institute of Health, 84 Val Fleuri, L-1526 Luxembourg

3: Present address: Lonza Cologne GmbH, Köln, Germany

4: Present address: Center for Retrovirus Research, Department of Veterinary Biosciences, The Ohio State University, Columbus, OH, USA

5: Department of Biomedicine, University of Bergen, Bergen, Norway

6: Department of Pathology, Haukeland University Hospital, Norway

7:Quantitative Biology Unit, , Luxembourg Institute of Health, Luxembourg

8: Present address: Genomics England, London, United Kingdom.

9: NorLux Neuro-Oncology Laboratory, Department of Oncology, Luxembourg Institute of Health, L-1526, Luxembourg, Luxembourg

10: nCounter Core Facility, Institute of Human Genetics, University of Heidelberg, 69120 Heidelberg, Germany

11: Department of Human Molecular Genetics, University of Heidelberg, 69120 Heidelberg, Germany

12: Institut de Génétique et de Biologie Moléculaire et Cellulaire (IGBMC), Illkirch, France

[*]Correspondence: *e-mails*: antonio.marchini@lih.lu; a.marchini@dkfz.de

Supplementary Figures 1 - 13
Supplementary Tables 1 – 3

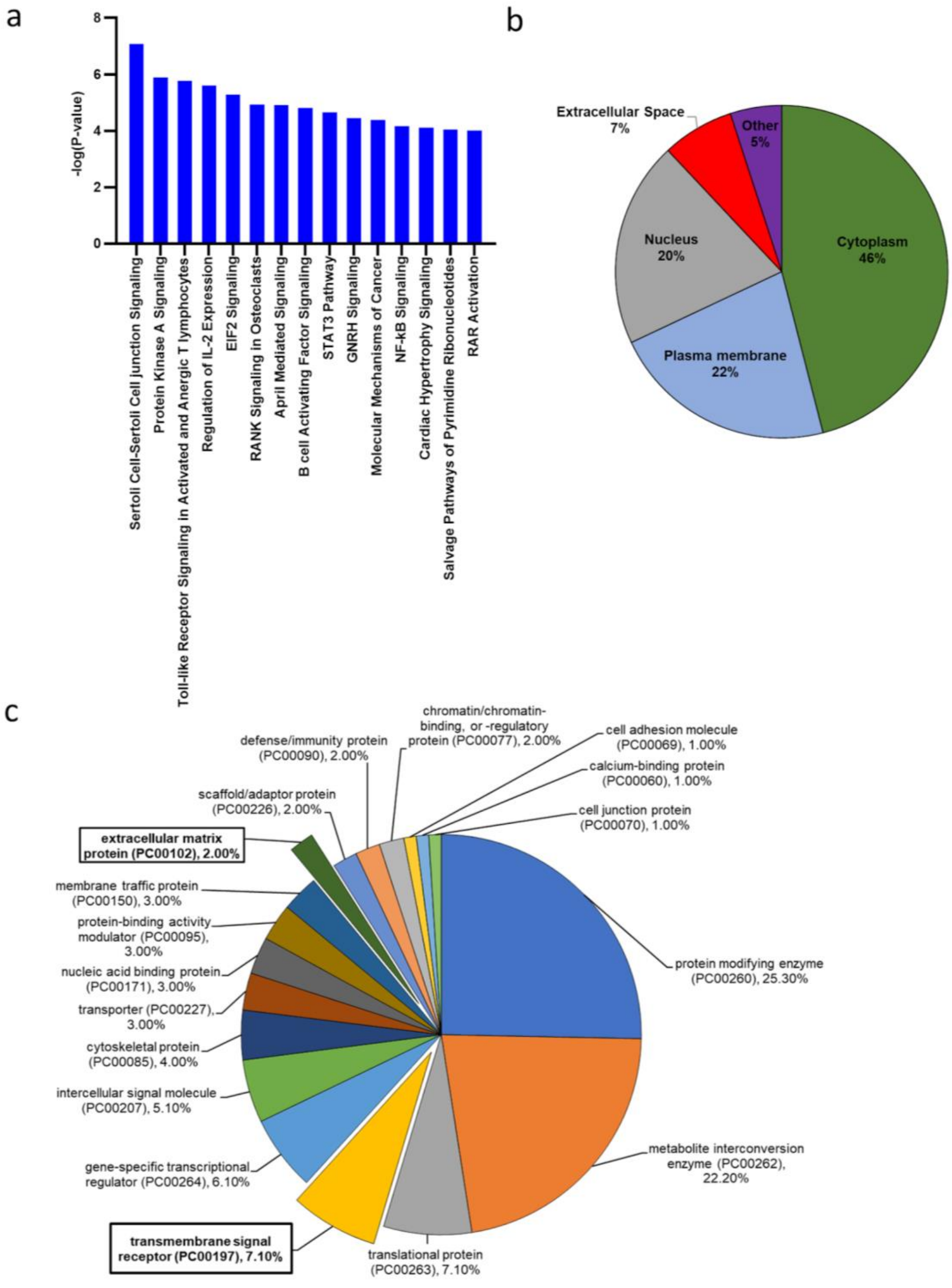

Supplementary Figure 1. Gene ontology enrichment analysis of the H-1PV activators identified by the siRNA library screening.

a, Ingenuity pathway analysis. Activators were classified based on their known participation in canonical pathways. The x-axis shows the top 15 canonical pathways, as tagged in the Ingenuity Knowledge Base, and the y-axis represents them as -log(P-value) based on the right-tailed Fisher's exact test. These pathways could possibly play a role in the H-1PV life cycle.

b, Cellular component gene ontology (GO) analysis. The top 151 H-1PV activators were classified based on their subcellular location according to the GO Knowledge Base.

c, GO PANTHER analysis. H-1PV activators were classified based on their cellular function according to the PANTHER Classification System.

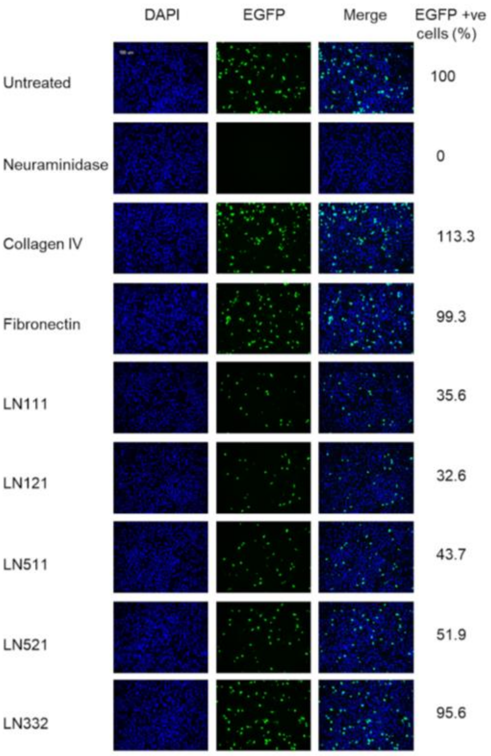

Supplementary Figure 2. Treatment with soluble laminins impairs H-1PV cell transduction.
HeLa cells were pre-incubated with indicated laminins (LN) or fibronectin or collagen and then infected with recH-1PV-EGFP. Neuraminidase was used as a positive control for blocking virus infection. Cells were then processed as described in Fig. 2c. As expected, neuraminidase treatment completely abolished H-1PV transduction, confirming that SA is required for H-1PV infection. Incubation with collagen and fibronectin did not decrease H-1PV transduction. Treatment with soluble laminins containing the γ1 chain all decreased H-1PV transduction, with some laminins (e.g., LN421 or LN221 in Fig. 3b) having a stronger effect than others (e.g. LN521). By contrast, LN332, which contains the γ2 chain, did not affect H-1PV transduction. Scale bar = 100 µm.

a

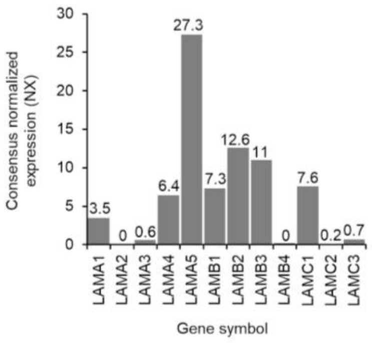

b

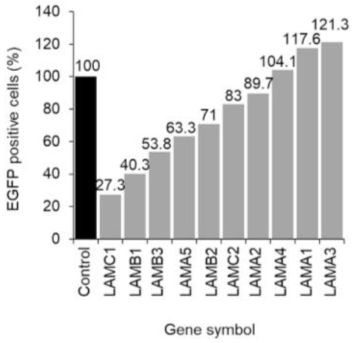

Supplementary Figure 3. Laminins are involved in H-1PV infection.

a, Consensus normalized mRNA transcript expression (NX) of all known laminins in HeLa. Values were obtained from the Human Protein Atlas. A value of NX < 1 indicates that an RNA transcript for the target gene was not detected in HeLa.

b, Indicated are the siRNA pools targeting laminin chains present in the siRNA library used for the screening (Fig. 1). Results are presented as the percentage of EGFP signal normalized to control siRNA, with values indicated on top of the columns. Several of these siRNA pools decrease H-1PV transduction.

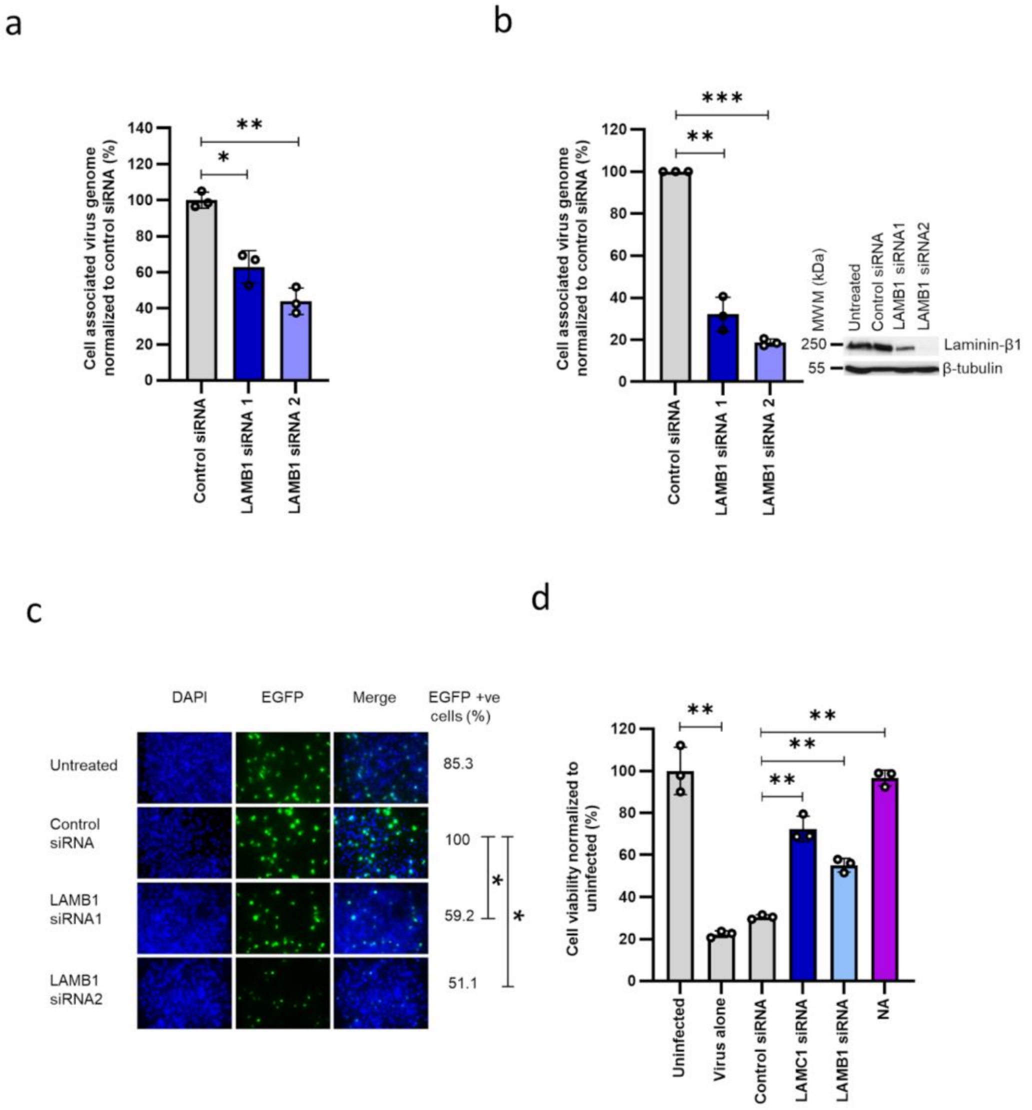

Supplementary Figure 4. Silencing of *LAMB1* decreases H-1PV infectivity.

a, Silencing of *LAMB1* decreases H-1PV cell binding. The cell binding/entry assay was performed at 4 °C as described in Fig. 2a. The representative experiment shown in the figure is repeated twice; n = 3 biologically independent samples; Control siRNA vs LAMB1 siRNA1 (P = 0.0126) and control siRNA vs LAMB1 siRNA2 (P = 0.0072).

b, Silencing of *LAMB1* decreases H-1PV cell uptake. At 46 h post-transfection with control siRNA or *LAMB1* siRNA, HeLa cells were infected with H-1PV for 4 h at 37 °C. Western blot analysis confirmed the siRNA-mediated down-regulation of *LAMB1* using β-tubulin as a loading control. Transfection of both siRNAs strongly decreased H-1PV cell entry. Scale bar = 100 μm. The average of three independent experiments shown in the figure; n = 2 biologically independent samples for each experiment; Control siRNA vs LAMB1 siRNA1 (P = 0.0047) and control siRNA vs LAMB1 siRNA2 (P = 0.0001).

c, Down-regulation of *LAMB1* by siRNAs decreases H-1PV transduction. At 46 h post-transfection with control siRNA or *LAMB1* siRNA, HeLa cells were infected with recH-1PV-EGFP for 24 h. The reduction of H-1PV transduction was observed with both LAMB1 siRNAs, thereby confirming the results obtained from the siRNA library screening.

d, Downregulation of *LAMB1* and *LAMC1* protects cells from H-1PV oncotoxicity. HeLa cells were transfected with control siRNA or siRNA targeting *LAMC1* or *LAMB1*, and then infected with H-1PV for 72 h. Neuraminidase treatment (NA) was used as a control. Cell viability was assessed by the CellTitre-Glo 2.0 assay. NA treatment completely protected the cells from virus-induced oncotoxicity. Cells transfected with siRNAs targeting *LAMC1* or *LAMB1* were less susceptible to virus oncotoxicity which correlated well with the decrease in virus binding/entry and transduction observed in these cells. The representative experiment shown in the figure is repeated twice; n = 3 biologically independent samples. Uninfected vs virus alone (P = 0.0061);

Control siRNA vs LAMC1 siRNA (P = 0.005); Control siRNA vs LAMB1 siRNA (P = 0.0074) and Control vs NA (P = 0.0017).
Statistical significance was calculated using a paired two-tailed t-test by GraphPad Prism 8 with *P< 0.05; **P< 0.01, ***P< 0.001. Error bars for all data indicate the mean values +/- SD. Source data are provided as Source Data file.

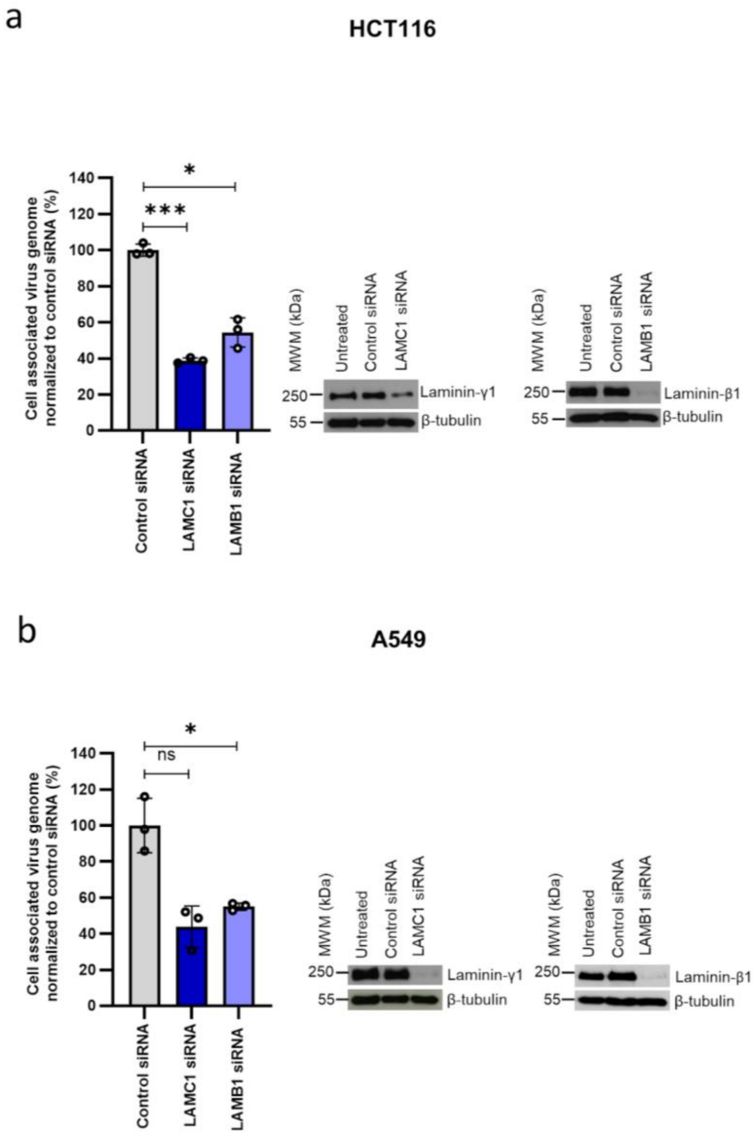

Supplementary Figure 5. *LAMC1* and *LAMB1* are involved in H-1PV virus cell uptake.
HCT116 (**a**) and A549 (**b**) cancer cell lines were used to confirm the results obtained in HeLa cells, which showed that the products of *LAMC1* and *LAMB1* are involved in H-1PV cellular uptake. Cells were transfected with indicated siRNAs and treated as described in Fig. 2b. The results are presented as percentage of virus genome associated with cells normalized to control siRNA. The independent experiment shown is repeated twice; n = 3 biologically independent samples. For HCT116 control siRNA vs LAMC1 siRNA ($P = 0.0004$) and control siRNA vs LAMB1 siRNA ($P = 0.0111$); For A549 control siRNA vs LAMC1 siRNA (P = ns) and control siRNA vs LAMB1 siRNA ($P = 0.0433$). Western blot analyses verified the siRNA-mediated down-regulation of the two genes using β-tubulin as a loading control. Statistical significance was calculated using a paired two-tailed t-test by GraphPad Prism 8 with *$P < 0.05$; **$P < 0.01$, ***$P < 0.001$; ns: not significant. Error bars for all data indicate the mean values +/- SD. Source data are provided as Source Data file.

a

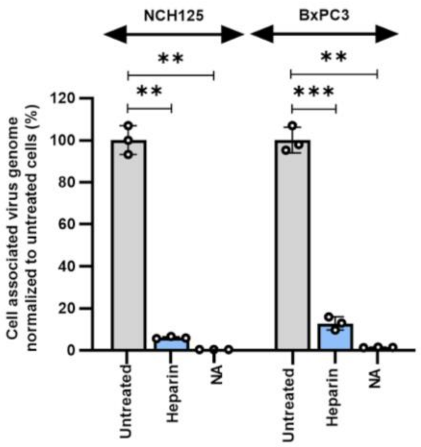

b

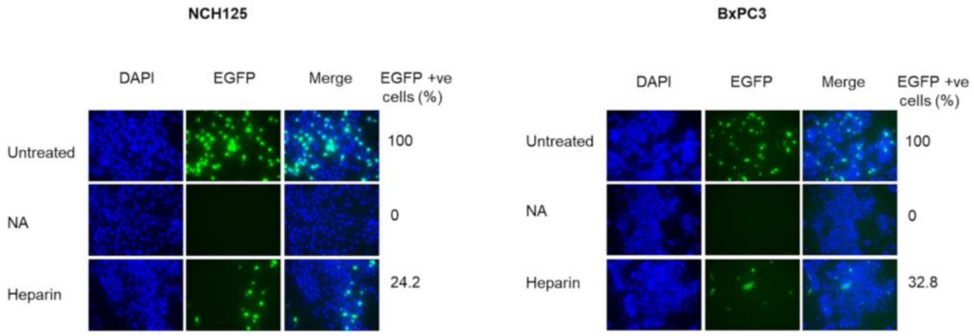

Supplementary Figure 6. Treatment with heparin impairs H-1PV cellular uptake.
a, Heparin impairs H-1PV uptake in glioma and pancreatic ductal adenocarcinoma (PDAC) cell lines. Both NCH125 (glioma) and BxPC3 (PDAC) cells were pre-incubated with heparin (100 µg/ml) for 24 h and then infected with H-1PV (MOI 1 pfu/cell) for 4 h at 37 °C. NA was used as a positive control for blocking virus cell binding/entry. Infection was completely blocked by NA, confirming the importance of SA for H-1PV cell binding/entry in these two cancer cell lines. Treatment with heparin also dramatically decreased H-1PV binding/entry. The independent experiment shown is repeated twice; n = 3 biologically independent samples. For NCH125 untreated vs heparin (P = 0.0015) and untreated vs NA (P = 0.0016); For BxPC3 untreated vs heparin (P = 0.0009) and untreated vs NA (P = 0.0013).
b, Treatment with heparin impairs H-1PV transduction in glioma and PDAC cell lines. Both NCH125 and BxPC3 were pre-incubated with heparin and then infected with recH-1PV-EGFP virus. At least 500 cells were analysed to calculate the percentage of EGFP-positive cells. Representative images are shown. Scale bar = 100 µm. Statistical significance was calculated using a paired two-tailed t-test by GraphPad Prism 8 with ***P< 0.001; ****P< 0.0001. Error bars for all data indicate the mean values +/- SD.
Source data are provided as Source Data file.

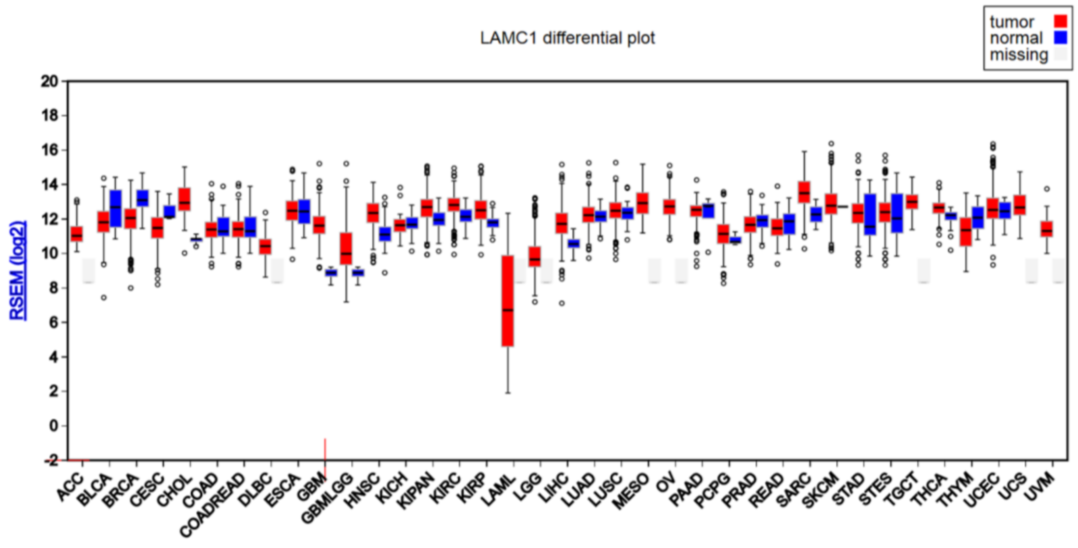

Cancer type abbreviations:

ACC	Adrenocortical carcinoma
BLCA	Bladder Urothelial Carcinoma
BRCA	Breast invasive carcinoma
CESC	Cervical squamous cell carcinoma and endocervical adenocarcinoma
CHOL	Cholangiocarcinoma
CNTL	Controls
COAD	Colon adenocarcinoma
DLBC	Lymphoid Neoplasm Diffuse Large B-cell Lymphoma
ESCA	Esophageal carcinoma
FPPP	FFPE Pilot Phase II
GBM	Glioblastoma multiforme
HNSC	Head and Neck squamous cell carcinoma
KICH	Kidney Chromophobe
KIRC	Kidney renal clear cell carcinoma
KIRP	Kidney renal papillary cell carcinoma
LAML	Acute Myeloid Leukemia
LCML	Chronic Myelogenous Leukemia
LGG	Brain Lower Grade Glioma
LIHC	Liver hepatocellular carcinoma
LUAD	Lung adenocarcinoma
LUSC	Lung squamous cell carcinoma
MESO	Mesothelioma
MISC	Miscellaneous
OV	Ovarian serous cystadenocarcinoma
PAAD	Pancreatic adenocarcinoma
PCPG	Pheochromocytoma and Paraganglioma
PRAD	Prostate adenocarcinoma
READ	Rectum adenocarcinoma
SARC	Sarcoma
SKCM	Skin Cutaneous Melanoma
STAD	Stomach adenocarcinoma
TGCT	Testicular Germ Cell Tumors
THCA	Thyroid carcinoma
THYM	Thymoma
UCEC	Uterine Corpus Endometrial Carcinoma
UCS	Uterine Carcinosarcoma
UVM	Uveal Melanoma

Supplementary Figure 7. Differential gene expression of *LAMC1* in tumours.
Box plots display gene expression in tumour (in red) vs. paired normal (in blue) samples across tissue sites. Information on missing data is also included (in grey). Data were obtained by consulting the public gene expression data viewer from Cancer Genome Atlas (TCGA) FireBrowse repository, http://firebrowse.org. The number of samples and fold change obtained for each tumour type and normal samples is accessed on interactive FireBrowse site by hovering cursor over the box in the plot. The horizontal line within the box indicates the median; boundaries of the box indicate the 1st and 3rd quartile; whiskers indicate the highest and lowest values.

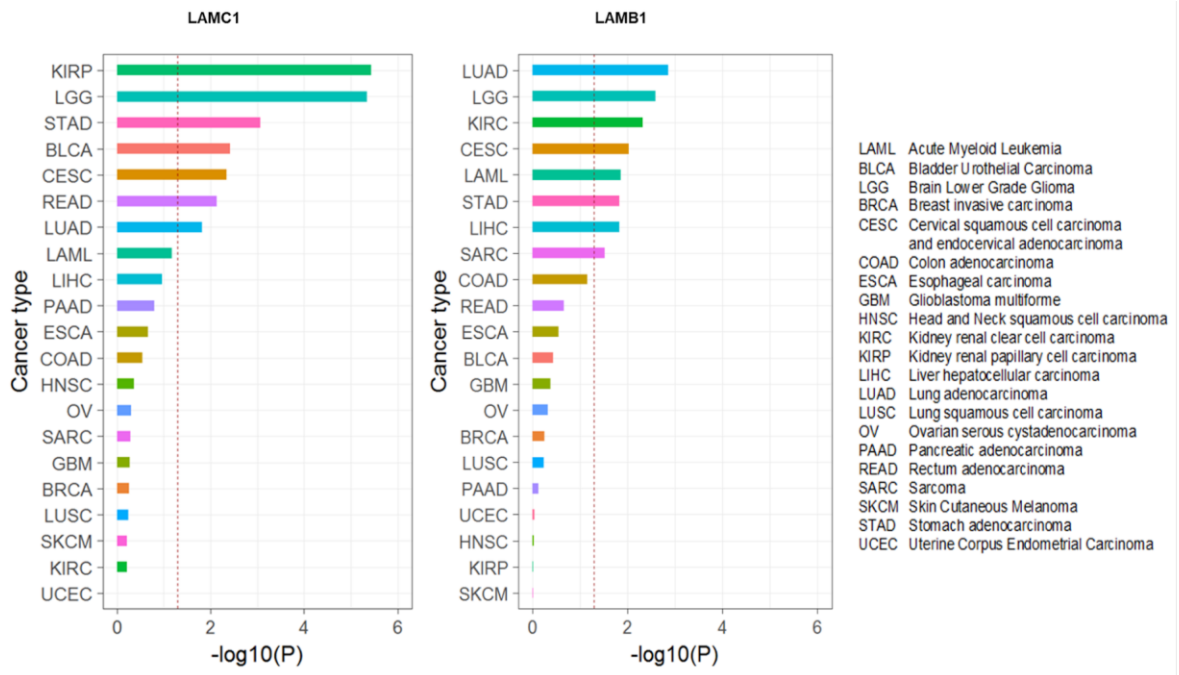

Supplementary Figure 8. *LAMC1* and *LAMB1* up-regulation is associated with poor prognosis in a variety of different cancers.

The gene expression data and clinical information from TCGA (http://cancergenome.nih.gov) was investigated using Cox regression models to explore the associations between *LAMC1* and *LAMB1* expression and patient survival in 21 cancer types. Both genes exhibited positive Cox regression coefficients: from 0.19 to 0.86 (LAMC1, in 7 cancer types), and from 0.19 to 0.36 (LAMB1, in 8 cancer types). These results indicate that the overexpression of these genes is associated with shorter median overall survival in patients with certain tumour types. In particular, *LAMC1* overexpression may be considered as a marker of poor prognosis in kidney renal papillary cell carcinoma (KIRP), brain lower grade glioma (LGG), stomach adenocarcinoma (STAD), bladder urothelial carcinoma (BLCA), cervical squamous cell carcinoma and endocervical adenocarcinoma (CESC), rectum adenocarcinoma (READ) and lung adenocarcinoma (LUAD) ($p < 0.05$). By contrast, *LAMB1* overexpression may be associated with poor prognosis in CESC, kidney renal clear cell carcinoma (KIRC), acute myeloid leukemia (LAML), LGG, liver hepatocellular carcinoma (LIHC), LUAD, sarcoma (SARC) and STAD ($p < 0.05$). To facilitate visualization, –log10 of the (Cox test) p-values are depicted on the x-axis for each cancer type (y-axis). The dotted red line indicates the position of $p = 0.05$.

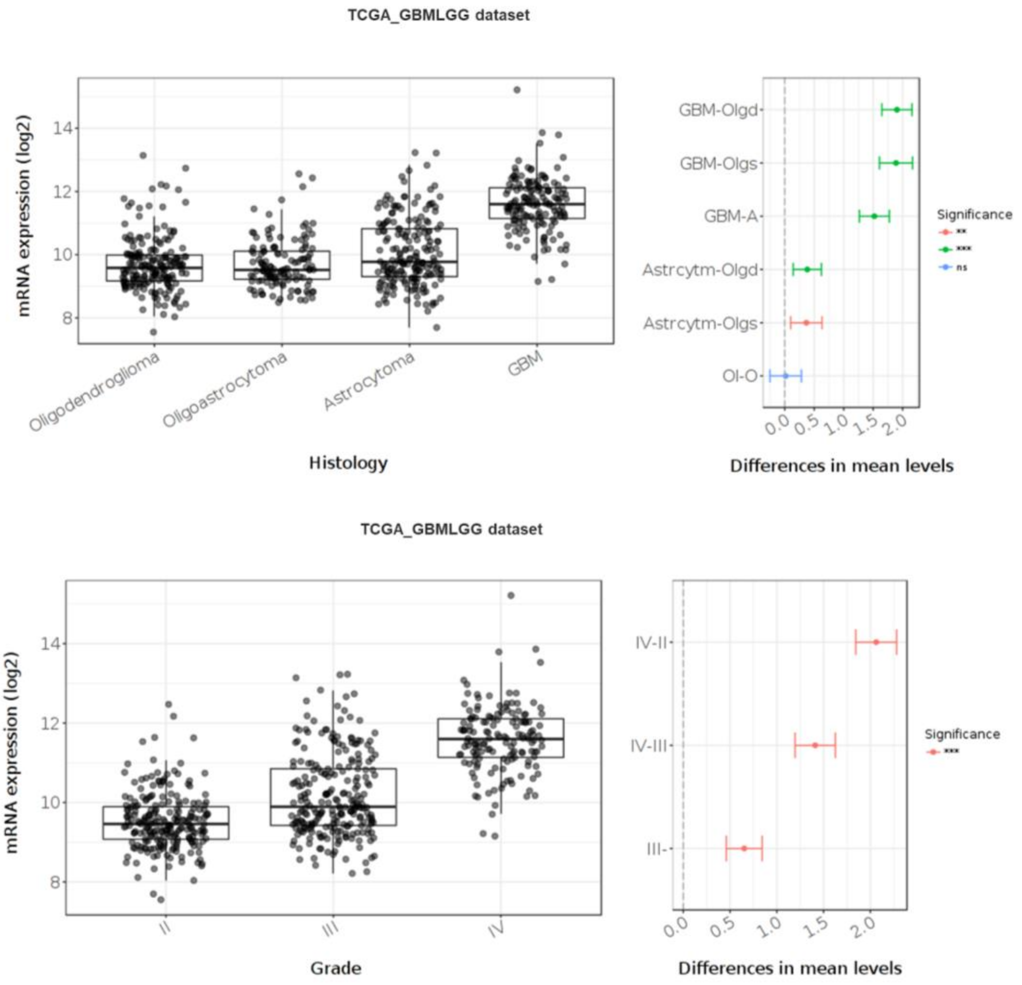

Supplementary Figure 9. ***LAMC1*** **is upregulated in glioblastoma in comparison to low-grade gliomas.** The interactive online GlioVis portal was used to analyse the differential expression of *LAMC1* among brain tumours using the TCGA_GBMLGG datasets obtained from RNA-Seq platform. Comparison of *LAMC1* expression across glioma grades and histopathological and molecular subtypes is shown. The Tukey's Honest Significant Difference (HDS) was used for statistical analysis. ***p<0.001; *p<0.05. The horizontal line within the box indicates the median, boundaries of the box indicate the 25th and 75th percentiles, and whiskers indicate the highest and lowest values.

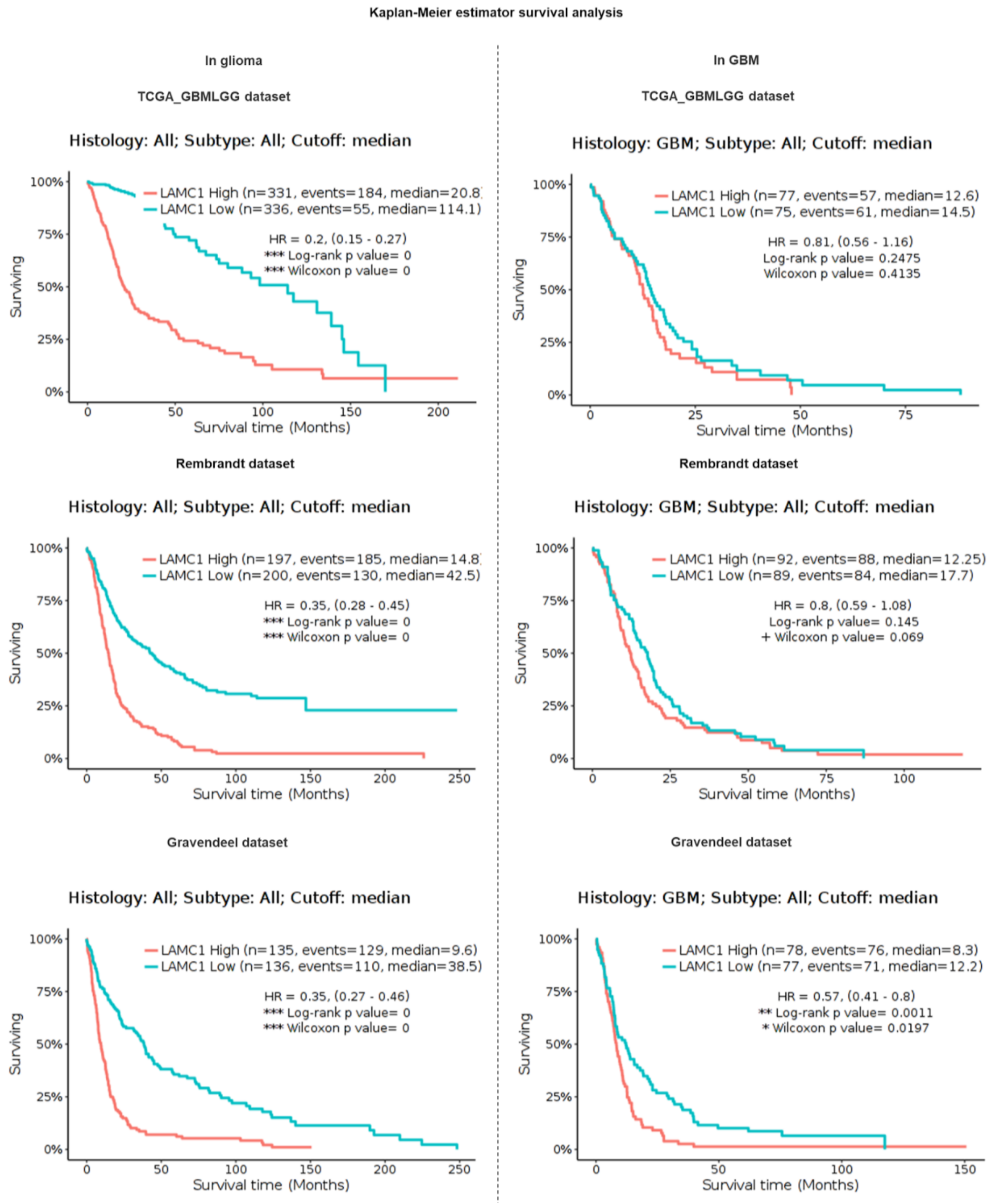

Supplementary Figure 10. ***LAMC1*** **over-expression is associated with poor prognosis in gliomas.** Kaplan-Meier analyses in glioma and GBM were performed using the GlioVis portal with the TCGA_GBMLGG, Rembrandt and Gravendeel datasets. Estimation of survival is based on tumour histology using the median expression levels of *LAMC1* as a cut-off. HR: hazard ratio.

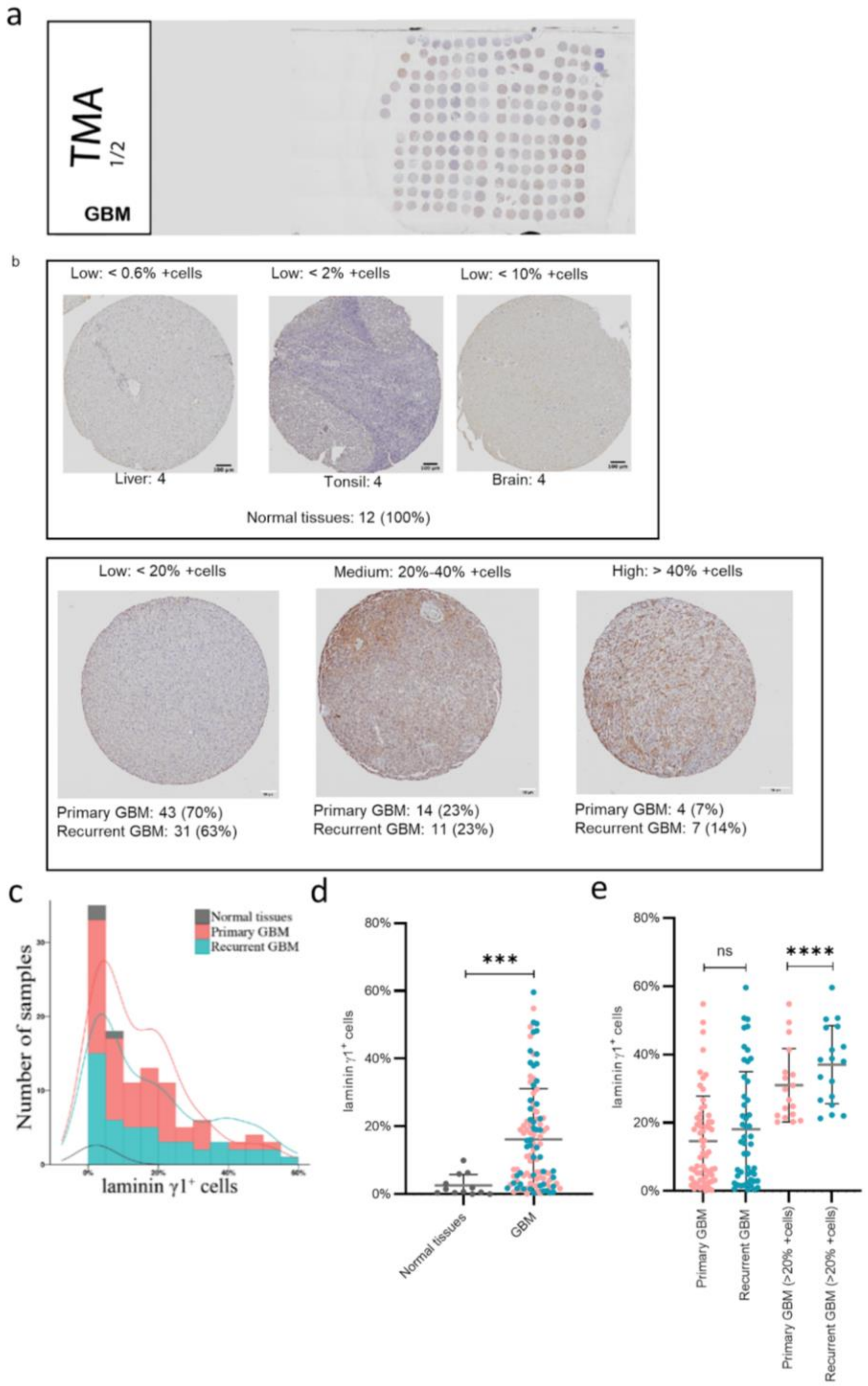

Supplementary Figure 11. Differential expression of laminin γ1 in normal tissues, primary GBM and recurrent GBM biopsies.

a, Overview of the immunostained tissue microarray (TMA). The TMA includes 110 different biopsies derived from primary (n=61) or recurrent (n=49) GBMs and 12 biopsies from normal tissues (liver, tonsil, and brain; each n = 4). TMA was stained with an anti-laminin γ1 chain antibody. The immunostaining was carried out on tumour samples in triplicates and on normal tissues in duplicates.

b, Representative images of biopsies with low, medium or high expression of laminin γ1 chain. Images were chosen for their closeness to the group average arbitrarily selected.

c, GBM biopsy distribution according to the number of laminin γ1 chain positive tumour cells (%). All normal tissues present low levels of laminin γ1 chain. Quantification was performed as described in the M&M section using software developed in-house.

d, Comparative analysis of laminin γ1 chain expression between normal tissues, and all 110 GBMs biopsies. $P = 0.0002$.
e, Comparative analysis of laminin γ1 expression between primary GBM, and recurrent GBM or their subpopulation with staining of laminin γ1 positive cells > 20%. Primary GBM vs recurrent GBM (P = ns); Primary GBM > 20% + cells vs recurrent GBM > 20% + cells ($P < 0.0001$).
Statistical significance was calculated using a paired two-tailed Student's t-test by GraphPad Prism 8 with ***$P< 0.001$; ****$P< 0.0001$; ns: not significant. Error bars for all data indicate the mean values +/- SD.

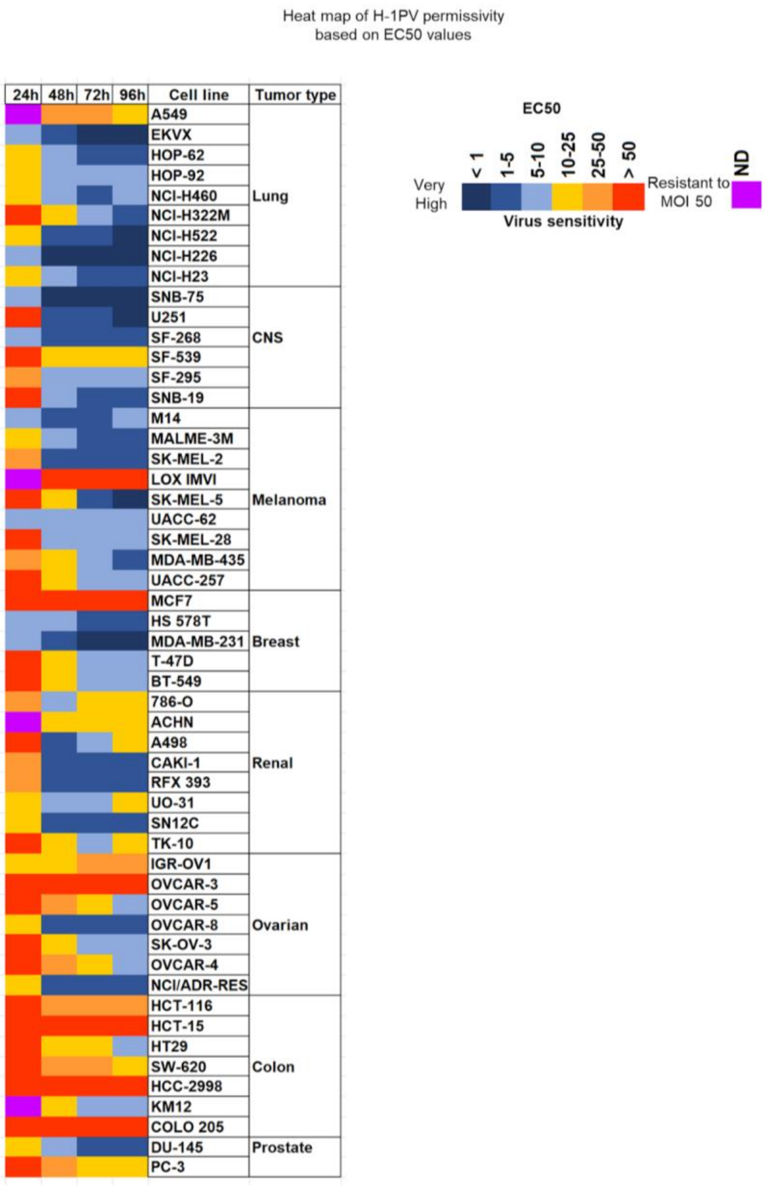

Supplementary Figure 12. Heat map of H-1PV sensitivity towards the NCI-60 cancer cell line panel. We screened 53 cancer cell lines from the NCI-60 cancer cell line panel for H-1PV sensitivity, as described in Figure 6. The heat map shows the EC50 values calculated for each cancer cell line at 24, 48, 72 and 96 h post-infection with H-1PV. Dark blue indicates very low EC50 values calculated in cell lines that were highly sensitive to H-1PV infection and killed by the lowest virus MOIs used (<1 pfu/cell). Dark red indicates very high EC50 values obtained in cancer cell lines that were resistant to the highest virus concentration used (MOI 50 pfu/cell). Intervening shades of blue, yellow and red indicate intermediate EC50 values. Purple indicates that the EC50 value was not determined (ND).

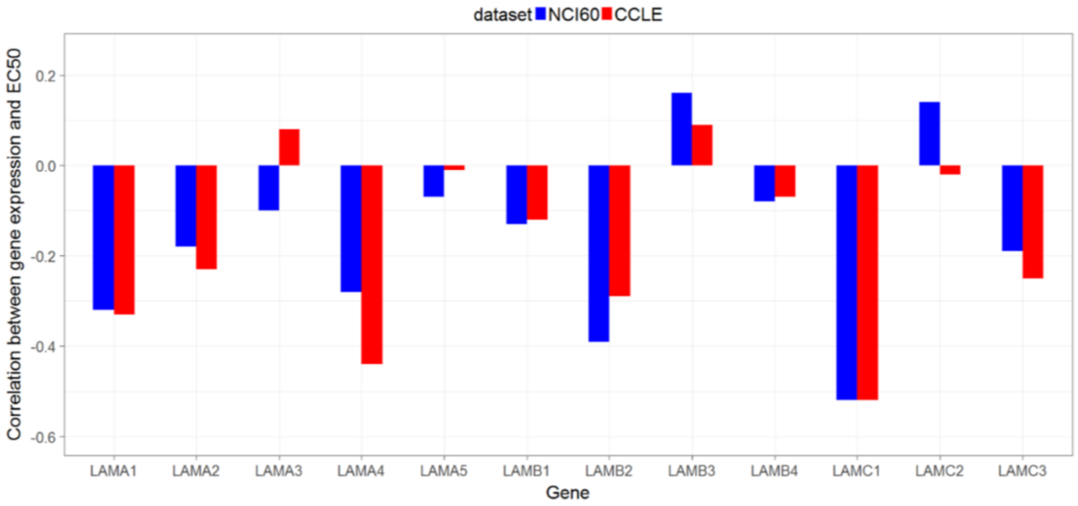

Supplementary Figure 13. Most laminin gene expression is robustly anticorrelated with EC50 values. Gene expression data from the NCI-60 (53 cell lines) and CCLE (38 cell lines) panels were anticorrelated with EC50 value (Pearson correlations). Bar plot shows the correlations, *r*, obtained in each dataset independently, together with *P* values (both with significant correlations at $P < 1E\text{-}3$, null hypothesis: $r = 0$).

Supplementary Table 1. List of H-1PV putative top activators belonging to the extracellular matrix protein and transmembrane signal receptor classes.

a) Extracellular matrix protein group

Gene symbol	Description	GFP signal (%)	Viability (%)	RNA expression level (NX) in HeLa
LAMC1	Laminin gamma 1 (formerly LAMB2)	27.33	97.2	7.6
LGALS1	Lectin galactoside-binding soluble 1	25.5	98.12	57.9

b) Transmembrane signal receptor group

Gene symbol	Description	GFP signal (%)	Viability (%)	RNA expression level (NX) in HeLa
CCKAR	Cholecystokinin receptor type A	14.29	85.48	0
TGFBR2	TGF-beta receptor type-2	17.26	119.88	1.6
ACVRL1	Serine/threonine-protein kinase receptor R3	23.57	94.35	0
DRD4	D(4) dopamine receptor	24.0	99.05	0.3
MC4R	Melanocortin receptor 4	27.5	107.4	0
IFNAR2	Interferon alpha/beta receptor 2	27.81	130.01	5.1
GPR92	Lysophosphatidic acid receptor 5	28.58	113.48	0.4

Note: The consensus normalized mRNA transcript expression levels (NX) of the indicated genes for the HeLa cell line were obtained from the Human Protein Atlas. A NX value < 1 indicates that mRNA transcripts for the target gene were not detected using mRNA sequencing technology.

Supplementary Table 2. The list of 53 cancer cell lines from the NCI-60 cancer cell lines panel screened for H-1PV sensitivity as described in Figure 6 and Supplementary Figure 12.

Tumour entity	NCI-60 cancer cell line
Lung	A549
	EKVX
	HOP-62
	HOP-92
	NCI-H460
	NCI-H322M
	NCI-H522
	NCI-H226
	NCI-H23
CNS	SNB-75
	U251
	SF-268
	SF-539
	SF-295
	SNB-19
Melanoma	M14
	MALME-3M
	SK-MEL-2
	LOX IMVI
	SK-MEL-5
	UACC-62
	SK-MEL-28
	MDA-MB-435
	UACC-257
Breast	MCF7
	HS 578T
	MDA-MB-231
	T-47D
	BT-549
Renal	786-O
	ACHN
	A498
	CAKI-1
	RFX 393
	UO-31
	SN12C
	TK-10
Ovarian	IGR-OV1
	OVCAR-3
	OVCAR-5
	OVCAR-8
	SK-OV-3
	OVCAR-4
	NCI/ADR-RES
Colon	HCT-116
	HCT-15
	HT29
	SW-620
	HCC-2998
	KM12
	COLO 205
Prostate	DU-145
	PC-3

Supplementary Table 3. List of primers/probe used in the study.

Primer/probe	Purpose	Sequence
LAMC1 For	Plasmid construction	5'-AAGAATATCAAGATCATGAGAGGGAGCCATCGGG-3'
LAMC1 Rev	Plasmid construction	5'-CGCCGAGGCCAGATCCTAGGGCTTTTCAATGGACGGG-3'
NS1 For	H-1PV DNA quantification	5′-GCGCGGCAGAATTCAAACT-3′
NS1 Rev	H-1PV DNA quantification	5′-CCACCTGGTTGAGCCATCAT-3′
NS1-TaqMan probe	H-1PV DNA quantification	5′-6-FAM-ATGCAGCCAGACAGTTA-MGB-3′

Supplementary Table 4. Target sequences and accession numbers of genes interrogated by nCounter expression profiling. *ACTB, GAPDH, and PGK1* were used as reference genes for expression data normalization.

Gene	Accession	Target Sequence
ACTB	NM_001101.2	TGCAGAAGGAGATCACTGCCCTGGCACCCAGCACAATGAAGATCAAGATCATTGCTCCTCCTGAGCGCAAGTACTCCGTGTGGATCGGCGGCTCCATCCT
GAPDH	NM_001256799.1	GAACGGGAAGCTTGTCATCAATGGAAATCCCATCACCATCTTCCAGGAGCGAGATCCCTCCAAAATCAAGTGGGGCGATGCTGGCGCTGAGTACGTCGTG
PGK1	NM_000291.2	GCAAGAAGTATGCTGAGGCTGTCACTCGGGCTAAGCAGATTGTGTGGAATGGTCCTGTGGGGGTATTTGAATGGGAAGCTTTTGCCCGGGGAACCAAAGC
LAMC1	NM_002293.3	TCTTGATAGGAAAGTGTCTGACCTGGAGAATGAAGCCAAGAAGCAGGAGGCTGCCATCATGGACTATAACCGAGATATCGAGGAGATCATGAAGGACATT
NS1	X01457.1	GCGGCAGAATTCAAACTAGAGAGGAGGTCTCGATTAAAACCACACTCAAAGAGTTGGTACATAAAAGAGTAACCTCACCAGAAGACTGGATGATGATGCA
