# Supplementary material for: Oncolytic H-1 parvovirus binds to sialic acid on laminins for cell attachment and entry
Source: Nat Commun. 2021 Jun 22;12:3834. doi: 10.1038/s41467-021-24034-7 (PMC8219832; doi:10.1038/s41467-021-24034-7)
Supplement: Supplementary file 3 — Reporting Summary [file 41467_2021_24034_MOESM3_ESM.pdf]

nature research

Corresponding author(s): Dr. Antonio Marchini

Last updated by author(s): 18/05/2021

Reporting Summary

Nature Research wishes to improve the reproducibility of the work that we publish. This form provides structure for consistency and transparency in reporting. For further information on Nature Research policies, see our Editorial Policies and the Editorial Policy Checklist.

Statistics

For all statistical analyses, confirm that the following items are present in the figure legend, table legend, main text, or Methods section.

n/a	Confirmed	
☐	☒	The exact sample size (*n*) for each experimental group/condition, given as a discrete number and unit of measurement
☐	☒	A statement on whether measurements were taken from distinct samples or whether the same sample was measured repeatedly
☐	☒	The statistical test(s) used AND whether they are one- or two-sided<br>*Only common tests should be described solely by name; describe more complex techniques in the Methods section.*
☒	☐	A description of all covariates tested
☒	☐	A description of any assumptions or corrections, such as tests of normality and adjustment for multiple comparisons
☐	☒	A full description of the statistical parameters including central tendency (e.g. means) or other basic estimates (e.g. regression coefficient) AND variation (e.g. standard deviation) or associated estimates of uncertainty (e.g. confidence intervals)
☐	☒	For null hypothesis testing, the test statistic (e.g. *F*, *t*, *r*) with confidence intervals, effect sizes, degrees of freedom and *P* value noted<br>*Give P values as exact values whenever suitable.*
☒	☐	For Bayesian analysis, information on the choice of priors and Markov chain Monte Carlo settings
☒	☐	For hierarchical and complex designs, identification of the appropriate level for tests and full reporting of outcomes
☐	☒	Estimates of effect sizes (e.g. Cohen's *d*, Pearson's *r*), indicating how they were calculated

Our web collection on statistics for biologists contains articles on many of the points above.

Software and code

Policy information about availability of computer code

Data collection	The IN Cell Investigator version 1.3 (GE Healthcare Life Sciences, Chicago, IL, USA), BZ-9000 fluorescence microscope (Keyence Corporation, USA), and IncuCyte ZOOM™ version 2018a live cell imaging system (Essen BioScience, Ann Arbor, MI, USA) softwares were used to acquire images.<br>The cell proliferation was monitored in real time using the RTCA-MT xCELLigence system version 1.2.1 (ACEA Biosciences Inc., San Diego, CA, USA).<br>The ELISA data was collected by Thermo Multiskan EX Microplate photometer software version 2.6 (Thermo Fisher).
Data analysis	The Multi Target Analysis module (IN Cell Investigator software version 1.3, GE Healthcare Life Sciences, Chicago, IL, USA) was used to segment cell nuclei (DAPI staining) and quantify EGFP co-localization to determine the percentage of transduced cell.<br>The single-cell data obtained from high-throughput cell imaging were analysed with the previously published RReportGenerator software version 1.3, which also determined statistical significance.<br>The annotation of genes (putative H-1PV activators) to a Ingenuity® Knowledge Base library of canonical pathways was carried out using Ingenuity® pathway analysis (IPA, Qiagen) software updated 2019.<br>The annotation of genes according to the cellular functions assigned by PANTHER (Protein ANnotation THrough Evolutionary Relationship) Protein Class ontology terms was carried out using PANTHER15.0 released version.<br>The analysis of colocalization was carried out using the ImageJ plug-in RGB profiler (https://imagej.nih.gov/ij/plugins/rgb-profiler.html) created by Christophe Laummonerie and Jerome Mutterer as described in the Materials section.<br>The analysis of cell proliferation of NCI-60 cancer cell lines were carried out using the RTCA-MT xCELLigence system version 1.2.1 (ACEA Biosciences Inc., San Diego, CA, USA).<br>The EC50 values (virus concentration killing 50% of cells) computation was conducted with the open-source statistical software environment R, version 2.14.2 (http://www.R-project.org) as described in Materials section.<br>The correlations between gene expression and EC50 analyses were calculated in R (Hmisc and ggplot2 libraries, Pearson correlation and corresponding P values).

The nCounter target gene expression analysis was carried out by NanoString Technologies (Seattle, WA, USA).
The normalization and evaluation of NanoString data was carried out using the nSolver Analysis Software (version 4.0) provided by NanoString Technologies (https://www.nanostring.com/products/analysis-software/nsolver).
The Gravendeel, Rembrandt and TCGA datasets for both target gene expression values and Kaplan-Meier patient survival estimation were analysed by means of the GlioVis data portal49 (http://gliovis.bioinfo.cnio.es/).
The statistical analysis of raw data was carried out using GraphPad Prism version 8 or Microsoft Excel 2016.

For manuscripts utilizing custom algorithms or software that are central to the research but not yet described in published literature, software must be made available to editors and reviewers. We strongly encourage code deposition in a community repository (e.g. GitHub). See the Nature Research guidelines for submitting code & software for further information.

Data

Policy information about availability of data

All manuscripts must include a data availability statement. This statement should provide the following information, where applicable:
- Accession codes, unique identifiers, or web links for publicly available datasets
- A list of figures that have associated raw data
- A description of any restrictions on data availability

All relevant data supporting the findings of this work are available within the paper and its Supplementary Information files. Source data are provided with this paper as a Source Data file. All other data are available from the corresponding author on request. Fig. 6c and d were generated employing the data sets publicly available at CellMiner™ (https://discover.nci.nih.gov/cellminer) and at The Cancer Cell Line Encyclopedia portals (https://portals.broadinstitute.org/ccle).
Supplementary Fig. 7 was generated employing The Cancer Genome Atlas (TCGA) FireBrowse, http://firebrowse.org. Supplementary Fig. 8 was generated using the gene expression data and clinical information from TCGA (http://cancergenome.nih.gov). Supplementary Figs. 9 and 10 were generated using the GlioVis data portal (http://gliovis.bioinfo.cnio.es/).

Field-specific reporting

Please select the one below that is the best fit for your research. If you are not sure, read the appropriate sections before making your selection.

☒ Life sciences ☐ Behavioural & social sciences ☐ Ecological, evolutionary & environmental sciences

For a reference copy of the document with all sections, see nature.com/documents/nr-reporting-summary-flat.pdf

Life sciences study design

All studies must disclose on these points even when the disclosure is negative.

Sample size	The sample size (n) of each experiment is indicated in the figure legends. No sample size calculation was performed. Sample size is chosen based on the standard of the corresponding field.
Data exclusions	No experimental data was excluded from the analysis.
Replication	All data shown derive from representative independent experiment with three replicates and repeated at least twice. Exception is ELISA experiment in which laminin trimers indicated in Fig. 5 are coated in duplicates for virus binding assay and independent experiment repeated twice and Nanostring experiment in which data shown correspond to the average of two technical replicates carried out for each biologically independent sample as mentioned in legends of Fig 6 e and f. Similar results were obtained from all repeated experiments.
Randomization	Randomization is not relevant to cell culture based experiments. The same number of cells were used for the control and treated experimental groups for comparing effect of treatment with the control.
Blinding	The experimental groups treatment was done in parallel as per sample procedures. The investigators who conducted the cell culture, qPCR and virus binding/entry assays, ELISA experiments were unblinded, since no bias would be introduced by the investigators. The sample preparation and analysis of Nanostring samples were done by two different investigators in a blinded manner. Investigators who performed siRNA library screening were blinded as well as those performing the protein microarray (protein quantification was performed by a different investigator).

Reporting for specific materials, systems and methods

We require information from authors about some types of materials, experimental systems and methods used in many studies. Here, indicate whether each material, system or method listed is relevant to your study. If you are not sure if a list item applies to your research, read the appropriate section before selecting a response.

Materials & experimental systems

n/a	Involved in the study
	☒ Antibodies
	☒ Eukaryotic cell lines
☒	☐ Palaeontology and archaeology
☒	☐ Animals and other organisms
☒	☐ Human research participants
☒	☐ Clinical data
☒	☐ Dual use research of concern

Methods

n/a	Involved in the study
☒	☐ ChIP-seq
☒	☐ Flow cytometry
☒	☐ MRI-based neuroimaging

Antibodies

Antibodies used

Laminin γ1 antibody (host: mouse, Commercial source and supplier name: Santa Cruz Biotechnology, catalog #: sc-13144, clone name: B-4)
Laminin γ1 antibody (host: mouse, Commercial source and supplier name: Santa Cruz Biotechnology, catalog #: sc-17751 clone name: D-3, lot #: L0904)
Laminin β1 antibody (host: rabbit, Commercial source and supplier name: Santa Cruz Biotechnology, catalog #: sc-5583 clone name: H-300)
β-tubulin (host: mouse, Commercial source and supplier name: Sigma-Aldrich Chemie GmbH, Steinheim, Germany, catalog #: T5201 clone name: TUB 2.1)
Pan-laminin antibody (host: rabbit, Commercial source and supplier name: Sigma-Aldrich Chemie GmbH, Steinheim, Germany, catalog #: L9393, lot #: 067M4872V)
Mouse monoclonal antibody IgG2a negative control (host: mouse, Commercial source and supplier name: EMD Millipore and Sigma-Aldrich Chemie GmbH, Steinheim, Germany, catalog #: MABC004 clone name: GC270, lot #: NG1853956)
Biotinylated anti-mouse IgG antibody (host: horse, Commercial source and supplier name: Vector Laboratories and Fisher scientific, catalog #: BA-2001, lot #: ZC1230)

Validation

Commercial primary antibodies were validated by the manufacturers and validation statements are available on the manufacturer`s websites.

Eukaryotic cell lines

Policy information about cell lines

Cell line source(s)

HeLa (human, Angel Alonso's laboratory-DKFZ, Heidelberg, Germany) Reference: Allaume X, El-Andaloussi N, Leuchs B, et al. Retargeting of rat parvovirus H-1PV to cancer cells through genetic engineering of the viral capsid. J Virol. 2012;86 (7):3452-3465. doi:10.1128/JVI.06208-11
HEK293T (human, ATCC)
NCH125 (human, Heidelberg University Hospital, Heidelberg, Germany) and
NCH37 (human, Heidelberg University Hospital, Heidelberg, Germany) Reference: Karcher S, Steiner HH, Ahmadi R, Zoubaa S, Vasvari G, Bauer H, Unterberg A, Herold-Mende C. Different angiogenic phenotypes in primary and secondary glioblastomas. Int J Cancer. 2006 May 1;118(9):2182-9. doi: 10.1002/ijc.21648. PMID: 16331629.
BxPC3 (human, DKFZ Tumor Bank, Heidelberg, Germany)
HCT116 (human, NCI, Rockville, MD, USA)
A549 (human, NCI, Rockville, MD, USA)
U251 (human, NCI, Rockville, MD, USA)
LN308 (human, Iris Augustin's laboratory-DKFZ, Heidelberg, Germany)
T98G (human, Iris Augustin's laboratory-DKFZ, Heidelberg, Germany)
A172-MG (human, Iris Augustin's laboratory-DKFZ, Heidelberg, Germany)
EKVX (human, NCI, Rockville, MD, USA)
HOP-62 (human, NCI, Rockville, MD, USA)
HOP-92 (human, NCI, Rockville, MD, USA)
NCI-H460 (human, NCI, Rockville, MD, USA)
NCI-H322M (human, NCI, Rockville, MD, USA)
NCI-H522 (human, NCI, Rockville, MD, USA)
NCI-H226 (human, NCI, Rockville, MD, USA)
NCI-H23 (human, NCI, Rockville, MD, USA)
SNB-75 (human, NCI, Rockville, MD, USA)
SF-268 (human, NCI, Rockville, MD, USA)
SF-539 (human, NCI, Rockville, MD, USA)
SF-295 (human, NCI, Rockville, MD, USA)
SNB-19 (human, NCI, Rockville, MD, USA)
M14 (human, NCI, Rockville, MD, USA)
MALME-3M (human, NCI, Rockville, MD, USA)
SK-MEL-2 (human, NCI, Rockville, MD, USA)

LOX IMVI (human, NCI, Rockville, MD, USA)
SK-MEL-5 (human, NCI, Rockville, MD, USA)
UACC-62 (human, NCI, Rockville, MD, USA)
SK-MEL-28 (human, NCI, Rockville, MD, USA)
MDA-MB-435 (human, NCI, Rockville, MD, USA)
UACC-257 (human, NCI, Rockville, MD, USA)
MCF7 (human, NCI, Rockville, MD, USA)
HS 578T (human, NCI, Rockville, MD, USA)
MDA-MB-231 (human, NCI, Rockville, MD, USA)
T-47D (human, NCI, Rockville, MD, USA)
BT-549 (human, NCI, Rockville, MD, USA)
786-O (human, NCI, Rockville, MD, USA)
ACHN (human, NCI, Rockville, MD, USA)
A498 (human, NCI, Rockville, MD, USA)
CAKI-1 (human, NCI, Rockville, MD, USA)
RFX 393 (human, NCI, Rockville, MD, USA)
UO-31 (human, NCI, Rockville, MD, USA)
SN12C (human, NCI, Rockville, MD, USA)
TK-10 (human, NCI, Rockville, MD, USA)
IGR-OV1 (human, NCI, Rockville, MD, USA)
OVCAR-3 (human, NCI, Rockville, MD, USA)
OVCAR-5 (human, NCI, Rockville, MD, USA)
OVCAR-8 (human, NCI, Rockville, MD, USA)
SK-OV-3 (human, NCI, Rockville, MD, USA)
OVCAR-4 (human, NCI, Rockville, MD, USA)
NCI/ADR-RES (human, NCI, Rockville, MD, USA)
HCT-116 (human, NCI, Rockville, MD, USA)
HCT-15 (human, NCI, Rockville, MD, USA)
HT29 (human, NCI, Rockville, MD, USA)
SW-620 (human, NCI, Rockville, MD, USA)
HCC-2998 (human, NCI, Rockville, MD, USA)
KM12 (human, NCI, Rockville, MD, USA)
COLO 205 (human, NCI, Rockville, MD, USA)
DU-145 (human, NCI, Rockville, MD, USA)
PC-3 (human, NCI, Rockville, MD, USA)
LAMC1-KD (human, In-house engineered HeLa with LAMC1 knock down, DKFZ, Heidelberg, Germany)
P3 (human, Haukeland University Hospital, Bergen, Norway)
P13 (human, Haukeland University Hospital, Bergen, Norway)

Authentication

All cell lines used in the study were obtained from and authenticated by vendors or scientific collaborators or by DKFZ cell authentication core facility.

Mycoplasma contamination

All the cell lines used in study were tested negative for mycoplasma contamination as evaluated by VenorGEM OneStep Mycoplasma contamination kit (Minerva Biolabs, Berlin, Germany).

Commonly misidentified lines
(See ICLAC register)

None
